# Mortality after Hospitalization for Pneumonia among Individuals with HIV, 1995–2008: A Danish Cohort Study

**DOI:** 10.1371/journal.pone.0007022

**Published:** 2009-09-14

**Authors:** Ole S. Søgaard, Nicolai Lohse, Jan Gerstoft, Gitte Kronborg, Lars Østergaard, Court Pedersen, Gitte Pedersen, Henrik Toft Sørensen, Niels Obel

**Affiliations:** 1 Department of Infectious Diseases, Aarhus University Hospital, Skejby, Denmark; 2 Department of Clinical Epidemiology, Aarhus University Hospital, Aarhus, Denmark; 3 Department of Infectious Diseases, Rigshospitalet, Copenhagen, Denmark; 4 Department of Infectious Diseases, Copenhagen University Hospital, Hvidovre, Denmark; 5 Department of Infectious Diseases, Odense University Hospital, Odense, Denmark; 6 Department of Infectious Diseases, Aarhus University Hospital, Aalborg, Denmark; University of New South Wales, Australia

## Abstract

**Background:**

HIV–infected persons are at increased risk of pneumonia, even with highly active antiretroviral treatment (HAART). We examined the impact of pneumonia on mortality and identified prognostic factors for death among HIV–infected.

**Methodology/Principal Findings:**

In a nationwide, population-based cohort of individuals with HIV, we included persons hospitalized with pneumonia from the Danish National Hospital Registry and obtained mortality data from the Danish Civil Registration System. Comparing individuals with and without pneumonia, we used Poisson regression to estimate relative mortality and logistic regression to examine prognostic factors for death following pneumonia. From January 1, 1995, to July 1, 2008, we observed 699 episodes of first hospitalization for pneumonia among 4,352 HIV patients. Ninety-day mortality after pneumonia decreased from 22.4% (95% confidence interval [CI]: 16.5%–28.9%) in 1995–1996 to 8.4% (95% CI: 6.1%–11.6%) in 2000–2008. Mortality remained elevated for more than a year after hospitalization for pneumonia: adjusted mortality rate ratio 5.38 (95% CI: 4.27–6.78), 1.80 (95% CI: 1.36–2.37), and 1.62 (95% CI: 1.32–2.00) for days 0–90, 91–365, and 366+, respectively. The following variables predicted mortality within 90 days following hospitalization for pneumonia (adjusted Odds Ratios): male sex (3.77, 95% CI: 1.37–10.4), Charlson Comorbidity Index score ≥2 (3.86, 95% CI: 2.19–6.78); no current HAART (3.58, 95% CI: 1.83–6.99); history of AIDS (2.46, 95% CI: 1.40–4.32); age per 10 year increase (1.43, 95% CI: 1.11–1.85); and CD4+ cell count ≤200 (2.52, 95% CI: 1.37–4.65).

**Conclusions/Significance:**

The first hospitalization for pneumonia among HIV–infected individuals was associated with elevated risk of death up to more than a year later. Use of HAART decreased the risk, independent of current CD4+ cell count. Prognosis following pneumonia improved over calendar time.

## Introduction

Early in the HIV epidemic it was recognized that morbidity and mortality due to pneumonia were higher in HIV-infected persons than in the general population [1]. In 1993 the United States Centers for Disease Control and Prevention categorized two or more episodes of bacterial pneumonia as an AIDS-defining event [2]. The introduction of highly active antiretroviral therapy (HAART) markedly reduced the incidence of AIDS and death among HIV-infected persons [3], [4], [5], and improved immune function also led to fewer pneumonia-related hospitalizations [6], [7]. However, more than a decade after the widespread introduction of HAART in high-income countries, the risk of pneumonia among HIV-infected persons remains high compared to persons without HIV [7]. A better understanding of modifiable prognostic factors for death after pneumonia could potentially reduce mortality from this illness. In this cohort study we estimated the impact of a first hospitalization for pneumonia on mortality among Danish HIV patients, examined changes over calendar time in mortality following hospitalization for pneumonia, and identified prognostic factors for death following pneumonia.

## Methods

### Study design and setting

We conducted a nationwide, population-based cohort study among HIV-infected persons in Denmark from 1995 to 2008. Treatment for HIV infection in Denmark is restricted to 8 specialized centers. The Danish health care system provides free, tax-supported medical care for all residents, including antiretroviral treatment of HIV.

### The Danish HIV Cohort Study (DHCS)

The DHCS has established a prospective, dynamic, nationwide, population-based cohort of all HIV-infected individuals seen in Danish HIV clinics since 1 January 1995. DHCS has been described in detail elsewhere [8], [9]. The study is ongoing, with continuous enrolment of both newly diagnosed patients and immigrants with HIV infection. Study data are updated annually with information on antiretroviral treatment, development of opportunistic infections and other AIDS-defining illnesses, and laboratory data including plasma HIV RNA (viral load (VL)) and CD4^+^ cell count.

### Danish Civil Registration System (CRS)

CRS is a national registry of all Danish residents, which contains information on date of birth, sex, date of migration, and date of death. A 10-digit personal registration number (CPR number), assigned at birth, uniquely identifies each person since 1968. The CRS is updated within a week of a person's birth, death, or emigration. Use of the CPR number enables Danish HIV clinics to avoid multiple registrations of the same patient and allows tracking of deaths and persons lost to follow-up due to emigration.

### The Danish National Hospital Registry (NHR)

NHR contains information on all patients discharged from Danish hospitals since 1977. Records for each hospitalization include CPR number, hospital department, inpatient and outpatient discharge diagnoses, and dates of admission and discharge. Diagnoses are coded by the treating physician according to the International Classification of Diseases, 8th revision (ICD-8) until the end of 1993 and 10th revision (ICD-10) thereafter.

### Identification of pneumonia

We identified the first hospitalization for pneumonia following HIV diagnosis. We used the NHR to identify all hospital stays with a discharge diagnosis of pneumonia using ICD-8 codes 471.x (influenza with pneumonia), 480.x-486.x (pneumonia), 073.x (ornithosis) and ICD-10 codes J11.0 (influenza with pneumonia), J12.x–J18.x (pneumonia), A481.x, (ornithosis), or A709.x (legionellosis). Thus, both community-acquired and hospital-acquired pneumonias were included. The pneumonia diagnoses recorded in the NHR were validated in a previous report [7]. Pneumonia onset was defined as the date of hospital admission. Since we have not validated pneumonia diagnoses in emergency room and outpatient settings, outpatient diagnoses were not included. AIDS-defining *Pneumocystis jiroveci* pneumonia was not counted as an episode of pneumonia.

### Study population

Our study population consisted of persons in DHCS who were at least 16 years old on the date of HIV diagnosis and who had no recorded hospitalization for pneumonia before entering DHCS. Study subjects were followed from their registration in DHCS to death, loss to follow-up or 1 July 2008, whichever came first.

### Definitions

*HAART* was defined as either a 3-drug regimen that included a non-nucleoside reverse transcriptase inhibitor, a protease inhibitor, and/or abacavir; or a 2-drug regimen with a combination of a non-nucleoside reverse transcriptase inhibitor and a boosted protease inhibitor.

*CD4^+^ cell counts and viral load (VL)* were estimated between measurements by carrying forward the value from the most recent measurement. *Nadir CD4^+^ cell count* was defined as the lowest CD4^+^ cell count ever measured for a given patient.

*Comorbidity* was assessed with the Charlson Comorbidity Index (CCI). The index, which includes 19 major disease categories, has been adapted and validated for use with hospital discharge data in ICD databases for predicting short- and long-term mortality [10]. A *CCI score* was computed for each patient based on all available foregoing NHR discharge diagnoses. A previous AIDS diagnosis (conferring 6 CCI points) was not included in our computations [11].

*Hepatitis C co-infection* was defined as patients having at least 1 positive result on a hepatitis C virus (HCV)-antibody test or a positive result on HCV RNA test.

The endpoint, defined a priori, was all-cause mortality following the hospital admission date for pneumonia. Causes of death, extracted from patient files and available in the DHCS database, were divided into HIV-related causes (AIDS-defining illnesses and bacterial infections, corresponding to ICD-10 codes A02, A07.2–07.3, A15–19, A31, A81.2, B00, B20–25, B37–39, B45, B58, C46, C53, C83.4,C83.9, F02.4, and J13–17 [pneumonia]), serious non–AIDS causes (cardiovascular disease [i.e. myocardial infarction or stroke], end stage renal and liver disease, COPD, and non-AIDS-defining malignancies), unnatural causes (i.e. drug overdose, suicide, accident) and other/unknown causes.

### Statistical analyses

We first computed 30-day and 90-day cumulative mortality following the first hospitalization for pneumonia and constructed Kaplan-Meier survival curves, stratified into 3 calendar periods: 1995–1996 (“pre-HAART era”), 1997–1999 (“early HAART era”), and 2000–2008 (“late HAART era”).

We then assessed the effect of a first hospitalization for pneumonia on mortality. We compared the mortality rate in persons who had no previous history of pneumonia (reference group) with that of persons with a first hospitalization for pneumonia within the last 0–90 days, within the last 91–365 days, and more than 365 days ago. Poisson regression analysis was used to adjust for potential confounders. The following time-dependent variables were forced into the model based on their presumed association with pneumonia and/or effect on mortality: CCI score (0–1/2+), age (10-year intervals), and CD4+ cell count (continuous). Other variables were examined and included in the final model if they changed the effect measure by 10% or more. Constant variables were sex (male/female), hepatitis C coinfection (yes/no), injection drug use (IDU) as presumed mode of HIV infection (yes/no), and race (Caucasian/non-Caucasian). Time-dependent variables were history of AIDS (yes/no), current HAART (yes/no), years since entering the DHCS, and calendar time period (1995–96 vs. 1997–2008). Causes of death were tabulated for all four time periods.

Finally, we used logistic regression to identify prognostic factors for 30-day and 90-day mortality following hospitalization for pneumonia. In the unadjusted analyses we included all the variables listed above as of the time of admission, as well as log-transformed HIV RNA (continuous). In the adjusted analyses of 30-day and 90-day mortality we included all variables from the unadjusted analyses in the models, except: nadir CD4+ cell count because this variable and history of AIDS are interdependent (adjustment for nadir CD4^+^ cell count thus could cancel out the effect of previous AIDS); IDU as mode of HIV exposure which is interdependent with hepatitis C status; and HIV RNA which is interdependent with use of HAART.

We used Stata software, version 9.2 (StataCorp, College Station, TX, USA) for statistical analyses. The study was approved by the Danish Data Protection Agency. In Denmark, a national board (The Danish Data protection Agency) approved the studies. Informed consent was waived. Informed consent was not required by Danish law in order to conduct cohort studies.

## Results

### DHCS study population

Between 1 January 1995 and 1 July 2008, 699 episodes of an initial hospitalization for pneumonia were observed among 4,352 persons who were at least 16 years old and had no recorded hospitalization for pneumonia before entering the DHCS cohort. Characteristics of persons in our study population at the time of the initial hospitalization for pneumonia are shown in Table 1. Less than half (43.3%) received HAART and the median CD4+ cell count was 281 cells/µl.

**Table 1:** Baseline charactaristics of HIV–infected individuals at time of first admission for pneumonia.

Variable		Numbers (N = 699)
Median age, years (interquartile range)		42.2 (35.2–50.1)
Sex, n (%)		
	Female	146 (20.9)
	Male	553 (79.1)
Race, n (%)		
	Caucasian	588 (84.2)
	Black	73 (10.5)
	Asian	12 (1.7)
	Inuit	10 (1.4)
	Other	15 (2.2)
Mode of HIV exposure, n (%)		
	MSM	299 (42.8)
	Heterosexual	204 (29.2)
	IV drug use	147 (21.0)
	Other	28 (4.0)
	Unknown	21 (3.0)
Hepatitis C co-infected, n (%)		
	Yes	191 (27.3)
	No	508 (72.7)
History of AIDS, n (%)		
	Yes	236 (33.8)
	No	463 (66.2)
Years since entering DHCS^a^, (interquartile range)		3.30 (1.18–7.01)
On HAART, n (%)		
	Yes	403 (57.7)
	No	296 (43.3)
Median current CD4+ cell count^b^, cells/µl (interquartile range)		281 (117–462)
Median days since last CD+ cell count measurement, (interquartile range)		47 (17–87)
Nadir CD4+ cell count, cells/µl (interquartile range)		139 (48–240)
Median HIV RNA^a^, log(copies/ml), (interquartile range)		
	On HAART	1.6 (1.3–4.1)
	HAART-naïve	4.6 (4.0–5.0)
Median days since last HIV RNA measurement, (interquartile range)		48 (19–85)
Charlson Comorbidity Index score, n (%)		
	0–1	532 (76.1)
	≥2	167 (23.9)

a DHCS: Danish HIV Cohort Study.

b Last measurement before date of pneumonia.

### Impact of hospitalization for pneumonia on survival among HIV patients

**Figure 1:**
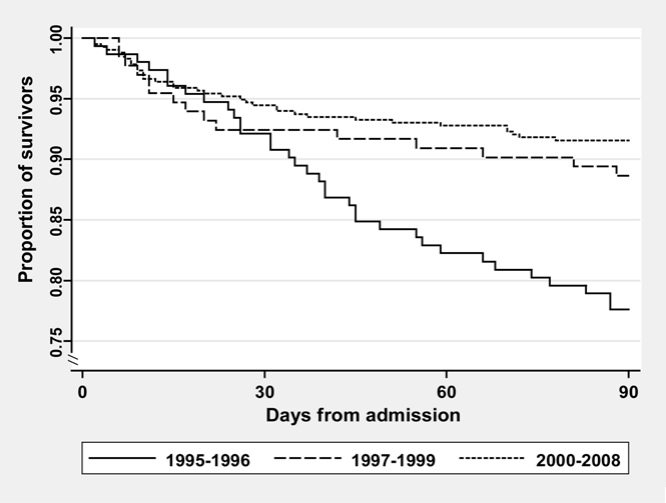
Mortality after first hospitalization for pneumonia among persons with HIV by time period.

Overall 30-day risk of death after first hospitalization for pneumonia was 6.4% (95% CI: 4.8%–8.5%) (see Figure 1). It was 7.9% (95% CI: 4.6%–13.5%) in 1995–1996, 7.6% (95% CI: 4.1%–13.6%) in 1997–1999, and 5.5% (95% CI: 3.7%–8.2%) in 2000–2008. Overall 90-day risk of death was 12.0% (95% CI: 9.8%–14.7%), decreasing from 22.4% (95% CI: 16.5%–28.9%) in 1995–1996 to 11.4% (95% CI: 7.0%–18.1%) in 1997–1999, and to 8.4% (95% CI: 6.1%–11.6%) in 2000–2008.

The effect of first-time hospitalization for pneumonia on mortality among all HIV-infected persons is shown in Table 2. Adjusting for use of HAART, history of AIDS, years since entering the DHCS, CCI score, age, and current CD4+ cell count, the relative mortality during the first 90 days after an initial hospitalization for pneumonia, compared to those with no previous hospitalization for this indication, was 5.38 (adjusted mortality rate ratio [MRR_adj_], 95% CI: 4.27–6.78). Mortality was also elevated for days 91–365 (MRR_adj_ = 1.80, 95% CI: 1.36–2.37) and days 366+ (MRR_adj_ = 1.62, 95% CI: 1.32–2.00). Causes of death for persons in all four time strata are presented in Table 3. Among those who died within days 0–90, days 91–365, and after 365 days, 61.9%, 53.7%, and 28.8%, respectively, had an HIV-related cause of death.

**Table 2:** Short- and long-term impact of pneumonia on mortality among all HIV–infected individuals.

	No of deaths	Follow-up^a^	MR^b^	Crude MRR ratio	Adjusted^c^ MRR
At any time after first hospitalization for pneumonia	263	3,131	0.23 (0.20–0.26)	3.56 (3.09–4.10)	2.79 (2.40–3.26)
Day 0–90 after admission	84	159	1.44 (1.17–1.79)	20.5 (16.3–25.7)	5.38 (4.27–6.78)
Day 91–365 after admission	54	420	0.35 (0.27–0.46)	4.98 (3.77–6.57)	1.80 (1.36–2.37)
Day 366+ after admission	125	2,552	0.13 (0.11–0.16)	1.90 (1.57–2.30)	1.62 (1.32–2.00)
No previous pneumonia	691	26,775	0.07 (0.07–0.08)	1 (ref)	1 (ref)

a In years.

b Per 1000 days.

c Adjusted for use of HAART, history of AIDS, years since entering the DHCS, Charlson Comorbidity Index score, age, and current CD4+ cell count.

**Table 3:** Causes of death among HIV patients with and without pneumonia.

	HIV-related^a^	Serious non-AIDS conditions^b^	Unnatural^c^	Other/unknown	No of deaths
Day 0–90 after pneumonia, n (%)	52 (61.9)	15 (17.9)	2 (2.4)	15 (17.9)	84 (100)
Day 91–365 after pneumonia, n (%)	29 (53.7)	7 (13.0)	3 (5.6)	15 (27.8)	54 (100)
Day 366+ after pneumonia, n (%)	36 (28.8)	36 (28.8)	5 (4.0)	48 (38.4)	125 (100)
No previous pneumonia, n (%)	296 (42.8)	145 (21.0)	42 (6.1)	208 (30.1)	691 (100)

a AIDS-defining illnesses and bacterial infections.

b Cardiovascular disease [i.e. myocardial infarction or stroke], end stage renal and liver disease, COPD, and non-AIDS-defining malignancies.

c I.e. drug overdose, suicide, and accident.

### Prognostic factors for short-term mortality following hospitalization for pneumonia

In the adjusted logistic regression analysis, the following variables were associated with increased 30-day mortality after a first hospitalization for pneumonia (Table 4): CCI score ≥2 (OR_adj_ = 4.07, 95% CI: 2.03–8.17), male sex (OR_adj_ = 3.86, 95% CI: 1.05–14.2), no current HAART (OR_adj_ = 3.19, 95% CI: 1.42–7.16), history of AIDS (OR_adj_ = 2.78, 95% CI: 1.34–5.78), latest CD4+ cell count ≤200 cells/µl (OR_adj_ = 2.72, 95% CI: 1.28–5.78) and age (OR_adj_ = 1.53 per 10 year increase, 95% CI: 1.11–2.12).

**Table 4:** Prognostics factors associated with 30-day mortality after first hospitalization for pneumonia among HIV–infected individuals.

			30 day mortality						
		n	Deaths	Mortality (%)	OR (95% CI)	p	OR_adj_ (95% CI)^a^	Coefficient (SE)	p
Intercept								−7.29 (1.11)	
Age (per 10 year increase)		699	45	6.4	1.47 (1.13–1.90)	0.004	1.53 (1.11–2.12)	0.43 (0.17)	0.010
CD4+ cell count^b^ <200 cells/µl	No	411	16	3.9	1 (ref)		1 (ref)	0 (ref)	
	Yes	241	27	11.1	3.11 (1.64–5.91)	0.001	2.72 (1.28–5.78)	1.00 (0.38)	0.009
	*missing data*	47	2	4.3	1.10 (0.24–4.93)	0.904	1.50 (0.29–7.74)	0.40 (0.84)	0.631
Charlson Comorbidty index	0–1	532	20	3.8	1 (ref)		1 (ref)	0 (ref)	
	≥2	167	25	15.0	4.51 (2.43–8.35)	<0.001	4.07 (2.03–8.17)	1.40 (0.36)	<0.001
History of AIDS	No	236	18	3.9	1 (ref)		1 (ref)	0 (ref)	
	Yes	463	27	11.4	3.19 (1.72–5.93)	<0.001	2.78 (1.34–5.78)	1.02 (0.37)	0.006
On HAART	Yes	347	17	4.9	1 (ref)		1 (ref)	0 (ref)	
	No	352	28	7.9	1.68 (0.90–3.12)	0.103	3.19 (1.42–7.16)	1.16 (0.41)	0.005
Sex	Female	146	3	2.0	1 (ref)		1 (ref)	0 (ref)	
	Male	553	42	7.6	3.92 (1.20–12.8)	0.024	3.86 (1.05–14.2)	1.35 (0.66)	0.042
Race	Non-caucasian	111	5	4.5	1 (ref)		1 (ref)	0 (ref)	
	Caucasian	588	40	6.8	1.55 (0.60–4.01)	0.369	0.47 (0.16–1.43)	−0.75 (0.57)	0.185
Calender period	1995–1996	152	12	7.9	1 (ref)		1 (ref)	0 (ref)	
	1997–2008	547	33	6.0	0.75 (0.38–1.49)	0.409	0.61 (0.23–1.57)	−0.50 (0.49)	0.305
Hepatitis C co-infected	No		31	6.1	1 (ref)		1 (ref)	0 (ref)	
	Yes		14	7.3	1.22 (0.63.2–34)	0.556	1.58 (0.74–3.39)	0.46 (0.39)	0.240
Mode of HIV exposure	Non-IDU	552	35	6.3	1 (ref)				
	IDU	147	10	6.8	1.08 (0.52–2.23)	0.839	…	…	…
		699	45	6.4	0.78 (0.61–0.99)	0.046	…	…	…
Nadir CD4+ cell count (per 100 cells/µl decrease)									
HIV RNA, (per log10 increase in copies/ml)^b^	On HAART	403	24	6.0	2.76 (0.40–19.2)	0.304	…	…	…
	HAART-naïve	296	21	7.1	0.36 (0.02.5.57)	0.468	…	…	…

a This logistic regression model is adjusted for all variables in the table except: IDU as mode of HIV exposure which is interdependent to hepatitis C status, Nadir CD4+ cell count which is interdependent to AIDS and HIV RNA which is interdependent to use of HAART.

b Last measurement before date of pneumonia. *adj* = Adjusted; OR = Odds Ratio; CI = Confidence Interval.

Variables associated with increased 90-day mortality were (Table 5): CCI score ≥2 (OR_adj_ = 3.86, 95% CI: 2.19–6.78), male sex (OR_adj_ = 3.77, 95% CI: 1.37–10.4), no current HAART (OR_adj_ = 3.56, 95% CI: 1.83–6.99), previous AIDS (OR_adj_ = 2.46, 95% CI: 1.40–4.32), CD4+ cell count ≤200 (OR_adj_ = 2.52, 95% CI: 1.37–4.65) and age (OR_adj_ = 1.43 per 10 year increase, 95% CI: 1.11–1.85). Among patients on HAART neither the duration of HAART use or HIV RNA (<50 vs. ≥50 copies/mL) were protective (data not shown).

**Table 5:** Prognostics factors for 90-day mortality after first hospitalization for pneumonia among HIV–infected individuals.

			90 day mortality						
		n	Deaths	Mortality (%)	OR (95% CI)	p	OR_adj_ (95% CI)^a^	Coefficient (SE)	p
Intercept								−6.82 (1.01)	
Age (per 10 year increase)		699	84	12.0	1.36 (1.11–1.66)	0.003	1.43 (1.11–1.85)	0.36 (0.13)	0.007
CD4+ cell count^b^ <200 cells/µl	No	411	28	6.8	1 (ref)		1 (ref)	0 (ref)	
	Yes	241	49	20.3	3.49 (2.13–5.73)	<0.001	2.52 (1.37–4.65)	0.93 (0.31)	0.003
	*missing data*	47	7	14.9	2.39 (0.98–5.83)	0.055	1.91 (0.67–5.43)	0.65 (0.53)	0.223
Charlson Comorbidty index	0–1	532	44	8.2	1 (ref)		1 (ref)	0 (ref)	
	≥2	167	40	24.0	3.49 (2.18–5.59)	<0.001	3.86 (2.19–6.78)	1.35 (0.29)	<0.001
History of AIDS	No	236	35	7.6	1 (ref)		1 (ref)	0 (ref)	
	Yes	463	49	20.8	3.20 (2.01–5.11)	<0.001	2.46 (1.40–4.32)	0.90 (0.29)	0.002
On HAART	Yes	347	25	7.2	1 (ref)		1 (ref)	0 (ref)	
	No	352	59	16.8	2.59 (1.58–4.25)	<0.001	3.58 (1.83–6.99)	1.27 (0.34)	<0.001
Sex	Female	146	5	3.4	1 (ref)		1 (ref)	0 (ref)	
	Male	553	79	14.3	4.40 (1.79–10.9)	0.001	3.77 (1.37–10.4)	1.33 (0.52)	0.010
Race	Non-caucasian	111	5	4.5	1 (ref)		1 (ref)	0 (ref)	
	Caucasian	588	79	13.4	3.29 (1.30–8.32)	0.012	1.15 (0.41–3.24)	0.14 (0.53)	0.791
Calender period	1995–1996	152	34	22.4	1 (ref)		1 (ref)	0 (ref)	
	1997–2008	547	50	9.1	0.35 (0.22–0.56)	<0.001	0.80 (0.39–1.64)	−0.22 (0.36)	0.548
Hepatitis C co-infected	No	508	63	12.4	1 (ref)		1 (ref)	0 (ref)	
	Yes	191	21	11.0	0.87 (0.52–1.47)	0.610	1.13 (0.61–2.11)	0.12 (0.32)	0.701
Mode of HIV exposure	Non-IDU	552	66	12.0	1 (ref)				
	IDU	147	8	12.3	1.03 (0.59–1.79)	0.924	…	…	…
Nadir CD4+ cell count (per 100 cells/µl decrease)		699	84	12.0	1.38 (1.13–1.69)	0.002	…	…	…
HIV RNA, (per log10 increase in copies/ml)^b^	On HAART	403	39	9.2	2.27 (0.43–12.1)	0.337	…	…	…
	HAART-naïve	296	45	14.9	0.24 (0.03–2.18)	0.206	…	…	…

a This logistic regression model was adjusted for all variables in the table except: IDU as mode of HIV exposure which is interdependent to hepatitis C status, Nadir CD4+ cell count which is interdependent to AIDS and HIV RNA which is interdependent to use of HAART.

b Last measurement before date of pneumonia. *adj* = Adjusted; OR = Odds Ratio; CI = Confidence Interval.

## Discussion

This study found that short-term mortality after a first hospitalization for pneumonia among HIV-infected individuals decreased from the pre-HAART to the late-HAART era. Despite this decrease over time, an episode of hospitalization due to pneumonia remains associated with an increased mortality among HIV patients. Prognostic factors were male sex, age, pre-existing comorbidity, low CD4 cell count, older age, and absence of HAART treatment. Several studies have shown that persons on HAART have a reduced risk of pneumonia compared to those not on HAART [6], [7], [12]. However, our study is the first to show that HAART use also affects prognosis in the presence of a pneumonia-related hospitalization.

The strengths of our study include use of a population-based, nationwide cohort with nearly complete inclusion and minor loss to follow-up; access to complete hospitalization data and vital statistics; and availability of electronically collected longitudinal data on viral load and CD4+ cell counts. The quality of the data minimized selection and information biases. Because we considered only the first hospitalization for pneumonia, our estimates were not biased by multiple pneumonia episodes occurring in highly susceptible individuals.

Our study had a number of limitations. First, previous studies have found identifiable bacterial pathogens in 24%–38% of HIV-infected patients with pneumonia [13], [14]. As data on specific pathogens were not available, we do not know the percentage of pneumonia cases in our study population caused by bacteria and how this may have affected prognosis. Second, causes of death registered in DHCS were based on information extracted from medical records. Thus the exact cause of death may be uncertain or, in some cases, multifactorial. For this reason we chose to group causes of death into broader categories to determine whether HIV was the key factor. We were unable to adjust for use of opportunistic infection prophylaxis, smoking status, and/or pneumococcal vaccination status since these data were not available in DHCS database. Our results are restricted to inpatients. Finally, despite our efforts to control for potential confounders, it is possible that the observed protective effect of HAART was due to confounding by indication (*i.e*., patients on HAART had an *a priori* reduced risk of death for example due to a healthier lifestyle compared to HAART-naïve patients).

The incidence rates of pneumonia in HIV-infected individuals decreased from 51 hospitalizations per 1000 person-years during 1995–1996 to 20 hospitalizations per 1000 person-years during 2005–2007 [7]. In our study population, 30-day mortality did not change over calendar time and was comparable to the mortality risk of 6%–8% following pneumonia found in other studies of HIV-infected persons [6], [13], and consistent with the finding that most pneumonia-related deaths occur within 30 days of hospital admission [15].

Contrary to studies on invasive pneumococcal disease [16], we found a decline in 90-day mortality from the pre-HAART era to the HAART era. We even may have underestimated the decline in mortality over calendar time because the median age in our cohort increased from 1995 to 2008. Therefore, while the acute course of pneumonia has change little over time, the reduction in 90-day mortality may be due to reduced risk of death from sequelae following the initial episode of pneumonia [17], perhaps stemming from a general improvement in immune function after introduction of HAART.

In a study of non-HIV infected individuals aged 40–64 hospitalized for the first time with pneumonia, 30-day mortality was 7.8%, and 90-day mortality was 11.6% [18]. These estimates are comparable to what we found in HIV-infected individuals. Further, the overall impact on risk of death following a first hospitalization pneumonia was similar to the increased risk recently reported for “mild” AIDS-defining events (*i.e.,* pulmonary and extrapulmonary tuberculosis, *pneumocystis jiroveci (carinii)* pneumonia and esophageal candidiases) [19].

Others have found a four to five-fold increased risk of death (follow-up ≤51 months) among HIV-infected persons with pneumonia, compared to those without pneumonia [6], [13], which is in accordance with our findings. Contrary to other studies [20], however, we found that the increased risk of death persisted beyond one year. Although unmeasured confounding factors cannot be ruled out as a contributory cause of the increased long term risk of death, pneumonia could also be viewed as a marker of immune system frailty that may not be reflected by the CD4+ cell count.

We found that HAART use improved the prognosis after a pneumonia-related hospitalization also after adjusting for CD4+ cell count, which is in line with results from earlier studies [21], [22], [23]. In a CD4+ cell-count-adjusted subgroup analysis from the Strategies for Management of Antiretroviral Therapy (SMART) trial, the risk of opportunistic diseases and death was increased in HAART-naïve patients or those off-HAART for 6 months compared to those with suppressed HIV viral load in plasma [21]. In an observational study of asymptomatic HIV patients with CD4+ counts of 351 to 500 cells/µl, deferring HAART vs. initiating early HAART was associated with a mortality ratio of 1.7 [23]. Brenchley and colleagues demonstrated that microbial translocation and immune activation were higher among HAART-naïve than those on HAART [24]. Whether increased immune activation influences an individual's ability to survive a serious infection such as pneumonia remains to be determined.

We observed a lower short term mortality among females than males. In the HIV Epidemiologic Research (HER) Study on pneumonia Kohli et al. found in-hospital mortality after bacterial pneumonia to be 7.7% among females [6], [7]. In our study, in which only 20.9% of the subjects were females, 30 day mortality was very low (2.0% compared to 7.6% for males). All-cause mortality rates may be moderately lower among females than males with HIV [4], but the considerable effect of gender on short term mortality found in our study may have been caused by chance and should be interpreted with caution.

In conclusion, a first hospitalization for pneumonia in HIV-infected individuals predicted an increased risk of death beyond one year. Among those hospitalized with pneumonia, use of HAART was associated with a reduced risk of death, independent of CD4 cell counts. While the former finding may indicate that acquisition of pneumonia is a marker of frailty, the latter may indicate that this frailty can be partly offset by use of HAART. These findings support the need for additional research to assess the role of HAART in reducing morbidity and mortality associated with non-AIDS-defining infections. Promotion of early HAART initiation may not only lead to reduced mortality after pneumonia, it may also reduce the risk of acquiring pneumonia severe enough to require hospitalization [6], [7]. Finally, it is reassuring that the prognosis following pneumonia has improved over calendar time.

## References

[1] Polsky B, Gold JW, Whimbey E, Dryjanski J, Brown AE (1986). Bacterial pneumonia in patients with the acquired immunodeficiency syndrome.. Ann Intern Med.

[2] (1992). 1993 revised classification system for HIV infection and expanded surveillance case definition for AIDS among adolescents and adults.. MMWR Recommendations and reports: Morbidity and mortality weekly report.

[3] Hogg RS, Heath KV, Yip B, Craib KJ, O'Shaughnessy MV (1998). Improved survival among HIV-infected individuals following initiation of antiretroviral therapy.. JAMA.

[4] Lohse N, Hansen AB, Pedersen G, Kronborg G, Gerstoft J (2007). Survival of persons with and without HIV infection in Denmark, 1995-2005.. Ann Intern Med.

[5] Palella FJ, Delaney KM, Moorman AC, Loveless MO, Fuhrer J (1998). Declining morbidity and mortality among patients with advanced human immunodeficiency virus infection. HIV Outpatient Study Investigators.. N Engl J Med.

[6] Kohli R, Lo Y, Homel P, Flanigan TP, Gardner LI (2006). Bacterial pneumonia, HIV therapy, and disease progression among HIV-infected women in the HIV epidemiologic research (HER) study.. Clin Infect Dis.

[7] Sogaard OS, Lohse N, Gerstoft J, Kronborg G, Ostergaard L (2008). Hospitalization for Pneumonia among Individuals With and Without HIV Infection, 1995-2007: A Danish Population-Based, Nationwide Cohort Study.. Clin Infect Dis.

[8] Obel N, Engsig FN, Rasmussen LD, Larsen MV, Omland LH (2008). Cohort Profile: The Danish HIV Cohort Study.. Int J Epidemiol.

[9] Lohse N, Hansen AB, Jensen-Fangel S, Kronborg G, Kvinesdal B (2005). Demographics of HIV-1 infection in Denmark: results from the Danish HIV Cohort Study.. Scand J Infect Dis.

[10] de Groot V, Beckerman H, Lankhorst GJ, Bouter LM (2003). How to measure comorbidity. a critical review of available methods.. J Clin Epidemiol.

[11] Charlson ME, Pompei P, Ales KL, MacKenzie CR (1987). A new method of classifying prognostic comorbidity in longitudinal studies: development and validation.. J Chronic Dis.

[12] Gordin FM, Roediger MP, Girard PM, Lundgren JD, Miro JM (2008). Pneumonia in HIV-infected persons: increased risk with cigarette smoking and treatment interruption.. Am J Respir Crit Care Med.

[13] Hirschtick RE, Glassroth J, Jordan MC, Wilcosky TC, Wallace JM (1995). Bacterial pneumonia in persons infected with the human immunodeficiency virus. Pulmonary Complications of HIV Infection Study Group.. N Engl J Med.

[14] Fine MJ, Stone RA, Singer DE, Coley CM, Marrie TJ (1999). Processes and outcomes of care for patients with community-acquired pneumonia: results from the Pneumonia Patient Outcomes Research Team (PORT) cohort study.. Arch Intern Med.

[15] Mortensen EM, Coley CM, Singer DE, Marrie TJ, Obrosky DS (2002). Causes of death for patients with community-acquired pneumonia: results from the Pneumonia Patient Outcomes Research Team cohort study.. Arch Intern Med.

[16] Grau I, Pallares R, Tubau F, Schulze MH, Llopis F (2005). Epidemiologic changes in bacteremic pneumococcal disease in patients with human immunodeficiency virus in the era of highly active antiretroviral therapy.. Arch Intern Med.

[17] Mortensen EM, Kapoor WN, Chang CC, Fine MJ (2003). Assessment of mortality after long-term follow-up of patients with community-acquired pneumonia.. Clin Infect Dis.

[18] Thomsen RW, Riis A, Norgaard M, Jacobsen J, Christensen S (2006). Rising incidence and persistently high mortality of hospitalized pneumonia: a 10-year population-based study in Denmark.. J Intern Med.

[19] Mocroft A, Sterne JA, Egger M, May M, Grabar S (2009). Variable impact on mortality of AIDS-defining events diagnosed during combination antiretroviral therapy: not all AIDS-defining conditions are created equal.. Clin Infect Dis.

[20] Kaplan V, Clermont G, Griffin MF, Kasal J, Watson RS (2003). Pneumonia: still the old man's friend?. Arch Intern Med.

[21] Emery S, Neuhaus JA, Phillips AN, Babiker A, Cohen CJ (2008). Major clinical outcomes in antiretroviral therapy (ART)-naive participants and in those not receiving ART at baseline in the SMART study.. J Infect Dis.

[22] Mocroft A, Ledergerber B, Katlama C, Kirk O, Reiss P (2003). Decline in the AIDS and death rates in the EuroSIDA study: an observational study.. Lancet.

[23] Kitahata MM, Gange SJ, Abraham AG, Merriman B, Saag MS (2009). Effect of Early versus Deferred Antiretroviral Therapy for HIV on Survival.. N Engl J Med.

[24] Brenchley JM, Price DA, Schacker TW, Asher TE, Silvestri G (2006). Microbial translocation is a cause of systemic immune activation in chronic HIV infection.. Nature Med.

